# RETRACTION: A Meta‐Analysis Examined the Effect of Topical Vancomycin Application in Decreasing Sternal Wound Infections Post Cardiac Surgery

**DOI:** 10.1111/iwj.70228

**Published:** 2025-02-11

## Abstract

**RETRACTION**: ZhangY. , ZhangP. , LiH. , ChiH. , ZhengN. , PanX. , and TangC. , “A Meta‐Analysis Examined the Effect of Topical Vancomycin Application in Decreasing Sternal Wound Infections Post Cardiac Surgery,” International Wound Journal 20, no. 6 (2023): 2068–2074, 10.1111/iwj.14074.36651221 PMC10333029

The above article, published online on 18 January 2023, in Wiley Online Library (http://onlinelibrary.wiley.com/), has been retracted by agreement between the journal Editor in Chief, Professor Keith Harding; and John Wiley & Sons Ltd. Following an investigation by the publisher, all parties have concluded that this article was accepted solely on the basis of a compromised peer review process. The editors have therefore decided to retract the article. The authors did not respond to our notice regarding the retraction.

**RETRACTION**: Y. Zhang , P. Zhang , H. Li , H. Chi , N. Zheng , X. Pan , and C. Tang , “A Meta‐Analysis Examined the Effect of Topical Vancomycin Application in Decreasing Sternal Wound Infections Post Cardiac Surgery,” International Wound Journal 20, no. 6 (2023): 2068–2074, 10.1111/iwj.14074.36651221 PMC10333029

The above article, published online on 18 January 2023, in Wiley Online Library (http://onlinelibrary.wiley.com/), has been retracted by agreement between the journal Editor in Chief, Professor Keith Harding; and John Wiley & Sons Ltd. Following an investigation by the publisher, all parties have concluded that this article was accepted solely on the basis of a compromised peer review process. The editors have therefore decided to retract the article. The authors did not respond to our notice regarding the retraction.

